# SMRT sequencing reveals differential patterns of methylation in two O111:H- STEC isolates from a hemolytic uremic syndrome outbreak in Australia

**DOI:** 10.1038/s41598-019-45760-5

**Published:** 2019-07-01

**Authors:** Brian M. Forde, Lauren J. McAllister, James C. Paton, Adrienne W. Paton, Scott A. Beatson

**Affiliations:** 1 Australian Infectious Diseases Centre, The University of Queensland, Brisbane, QLD Australia; 2 Australian Centre for Ecogenomics, School of Chemistry and Molecular Biosciences, The University of Queensland, Brisbane, QLD Australia; 3 Research Centre for Infectious Diseases, Department of Molecular and Biomedical Science, University of Adelaide, Adelaide, SA Australia

**Keywords:** Bacterial evolution, Bacterial genomics, Bacterial genetics

## Abstract

In 1995 a severe haemolytic-uremic syndrome (HUS) outbreak in Adelaide occurred. A recent genomic analysis of Shiga toxigenic *Escherichia coli* (STEC) O111:H- strains 95JB1 and 95NR1 from this outbreak found that the more virulent isolate, 95NR1, harboured two additional copies of the Shiga toxin 2 (Stx2) genes encoded within prophage regions. The structure of the Stx2-converting prophages could not be fully resolved using short-read sequence data alone and it was not clear if there were other genomic differences between 95JB1 and 95NR1. In this study we have used Pacific Biosciences (PacBio) single molecule real-time (SMRT) sequencing to characterise the genome and methylome of 95JB1 and 95NR1. We completely resolved the structure of all prophages including two, tandemly inserted, Stx2-converting prophages in 95NR1 that were absent from 95JB1. Furthermore we defined all insertion sequences and found an additional IS*1203* element in the chromosome of 95JB1. Our analysis of the methylome of 95NR1 and 95JB1 identified hemi-methylation of a novel motif (5′-CTGC^m6^AG-3′) in more than 4000 sites in the 95NR1 genome. These sites were entirely unmethylated in the 95JB1 genome, and included at least 177 potential promoter regions that could contribute to regulatory differences between the strains. IS*1203* mediated deactivation of a novel type IIG methyltransferase in 95JB1 is the likely cause of the observed differential patterns of methylation between 95NR1 and 95JB1. This study demonstrates the capability of PacBio SMRT sequencing to resolve complex prophage regions and reveal the genetic and epigenetic heterogeneity within a clonal population of bacteria.

## Introduction

In 1995 a large outbreak of hemolytic uremic syndrome (HUS) occurred in Adelaide, South Australia. This outbreak was attributed to uncooked, fermented, dry sausage contaminated with Shiga toxigenic *Escherichia coli* (STEC) O111:H-^1^. A total of 23 cases were confirmed, all in children aged 6 months - 14 years. As a result of infection sixteen of the 23 children required dialysis and one 4 year old child died. STEC isolates from the outbreak were found to be highly virulent with an infectious dose requiring as little as 1 organism per 10 g of sausage. Interestingly, strains isolated after January 25^th^ 1995 appeared more virulent with patients presenting with O111 STEC after this date experienced more severe disease (including the fatality)^2^. Based on Southern blot analysis, whereas early patient isolates (pre January 25^th^) had both *stx1* and *stx2* Shiga toxin genes, later patient isolates (post January 25^th^) were also predicted to encode a second copy of *stx*2AB^1,3^. This genetic difference was hypothesised to account for the afore-mentioned difference in virulence between isolates collected before and after January 25^th^ (represented by 95JB1 and 95NR1, respectively). Consistent with this hypothesis, the total Shiga toxin titre, measured as 50% cytotoxic doses (CD_50_)/mL, was four times higher in culture lysates of 95NR1 than 95JB1^4^.

Recently, the 1995 Adelaide outbreak was re-examined by sequencing the genomes of 95JB1 and 95NR1 on the Illumina GAII platform^4^. Comparison of the draft genomes of 95JB1 and 95NR1 identified a Stx1-converting prophage and Stx2-converting prophage shared by both strains as well as ~50 kb of phage-associated sequence that was present in 95NR1 but absent in 95JB1. Based on read coverage and long-range PCR it was inferred that there were two additional Stx2 prophage in 95NR1 when compared to 95JB1^4^. Consistent with a heterogeneous population of O111 STEC within the primary source for the contaminated sausage (such as a livestock herd or individual animal), all core genome single nucleotide polymorphisms (SNPs) differentiating 95JB1 from 95NR1 (and the O111 STEC reference strain 11128) had occurred in 95JB1^4^. These results indicated that 95JB1 was a derivative of 95NR1, even though 95JB1 was isolated earlier in the outbreak, and suggests that the two additional Stx2 prophages were likely deleted from 95JB1 rather than acquired by 95NR1^4^. However, due to the inability of short read sequencing technologies to accurately resolve repetitive loci, the structure and genomic context of these prophages could not be unambiguously resolved.

The Pacific Biosciences (PacBio) Single Molecule Real Time (SMRT) sequencing platform is able to completely resolve most bacterial genomes by producing reads of sufficient length to span complex repeat loci and generate complete assemblies without the need for costly manual finishing^5–9^. A remarkable feature of SMRT sequencing is the capacity to determine the methylation status of every sequenced nucleotide^10^. DNA methylation is the most common post replicative modification in bacteria^11^ and is known to influence a wide variety of host processes, including DNA replication, repair and transcriptional regulation^12^. Until recently the lack of a simple, efficient method to determine the methylation status of DNA has resulted in these epigenetic modifications being largely ignored in the bacteria.

Here we sought to examine if any genetic or epigenetic differences (other than Stx2 copy number) could potentially contribute to virulence differences between the STEC O111:H- outbreak strains 95JB1 and 95NR1. Using PacBio SMRT sequencing to determine their complete genome assemblies and methylomes, we completely resolve the genetic structure of all prophage-encoding regions, including two distinct, but closely related, Stx2-converting prophage in 95NR1 that are tandemly inserted in the same genomic location and appear to have been deleted from 95JB1. We identify all putative methyltransferases in both strains, define their target sequences and show the activity of a previously uncharacterised methyltransferase in 95NR1 that is not active in 95JB1. Furthermore, we unambiguously determine the copy number and context of all IS elements in the genomes of 95NR1 and 95JB1 and reveal that a single difference in the IS complement could be directly responsible for differences in their methylome profiles.

## Results

### Complete genome assembly reveals full sequence of tandemly arrayed Stx2 prophages in 95NR1

*De novo* assembly of *E. coli* O111:H- strains 95JB1 and 95NR1 generated single circular chromosomal contigs of 5,347,879 bp and 5,467,946 bp, respectively. Previously reported short read assemblies of 95NR1 (accession: AVDU00000000.1) and 95JB1 (accession: AWFJ00000000.1) contained 182 and 179 contigs, respectively, with contig N50 sizes of less than 100 kb^4^ (Table 1). Two plasmids, similar to the P1 and EHEC plasmids from *E. coli* 11128^13^, were completely assembled in the PacBio assemblies of 95JB1 and 95NR1 (p95NR1A/p95JB1A and p95NR1B/p95JB1B, respectively). In contrast to the draft Illumina assemblies of 95JB1 and 95NR1^4^, the small pO111_4 and pO111_5 colicin plasmids were not detected in the PacBio assemblies or in the raw PacBio read data consistent with their exclusion during the library preparation process.

**Table 1:** Assembly Statistics.

Strain	Number of SMRTCells	Number of PacBio contigs	PacBiocontig N50 (bp)	Number of Illumina Contigs	Illumina contig N50 (bp)
95JB1	2	4	5373164	179	91606
95NR1	2	3	5462770	182	94405

A SNP comparison of the complete genomes of 95JB1 and 95NR1 is in agreement with previous observations using Illumina data^4^. Using *E. coli* O111:H- strain 11128 as a reference, McAllister *et al*. identified six SNPs, 5 on the chromosome and one plasmid encoded SNP, which discriminate 95JB1 from 95NR1^4^. Our SNP comparison of the complete PacBio assemblies of 95JB1 and 95NR1 identified four of the five chromosomally located 95JB1 SNPs and the single plasmid encoded SNP (Table 2). The remaining chromosomal SNP was located in a gene encoding a phage tail protein and due to the repetitiveness of these genes it was not considered reliable for strain discrimination.

**Table 2:** SNPs differentiating 95NR1 and 95JB1.

Strain	Base Change	95JB1 site	95NR1 site	Amino Acid Change	Illumina^1^	PacBio	Annotation
95JB1	C-T	587038	587038	P-L	+	+	*glxK* (Glycerate kinase II
95JB1	G-T	3227026	3227373	V-F	+	+	End of Stx1 prophage
95JB1	G-A	3602596	3602945	E-K	+	+	*metK* (Methionine adenosyltransferase 1)
95JB1	G-C	3813994	3814343	Stop codon-Y	+	+	*fadH* (2, 4-dienoyl-CoA reductase)
p95JB1B	G-C	20482	20477	P-A	+	+	

^1^SNPs identified by McAllister *et al*.^4^.

**Figure 1:**
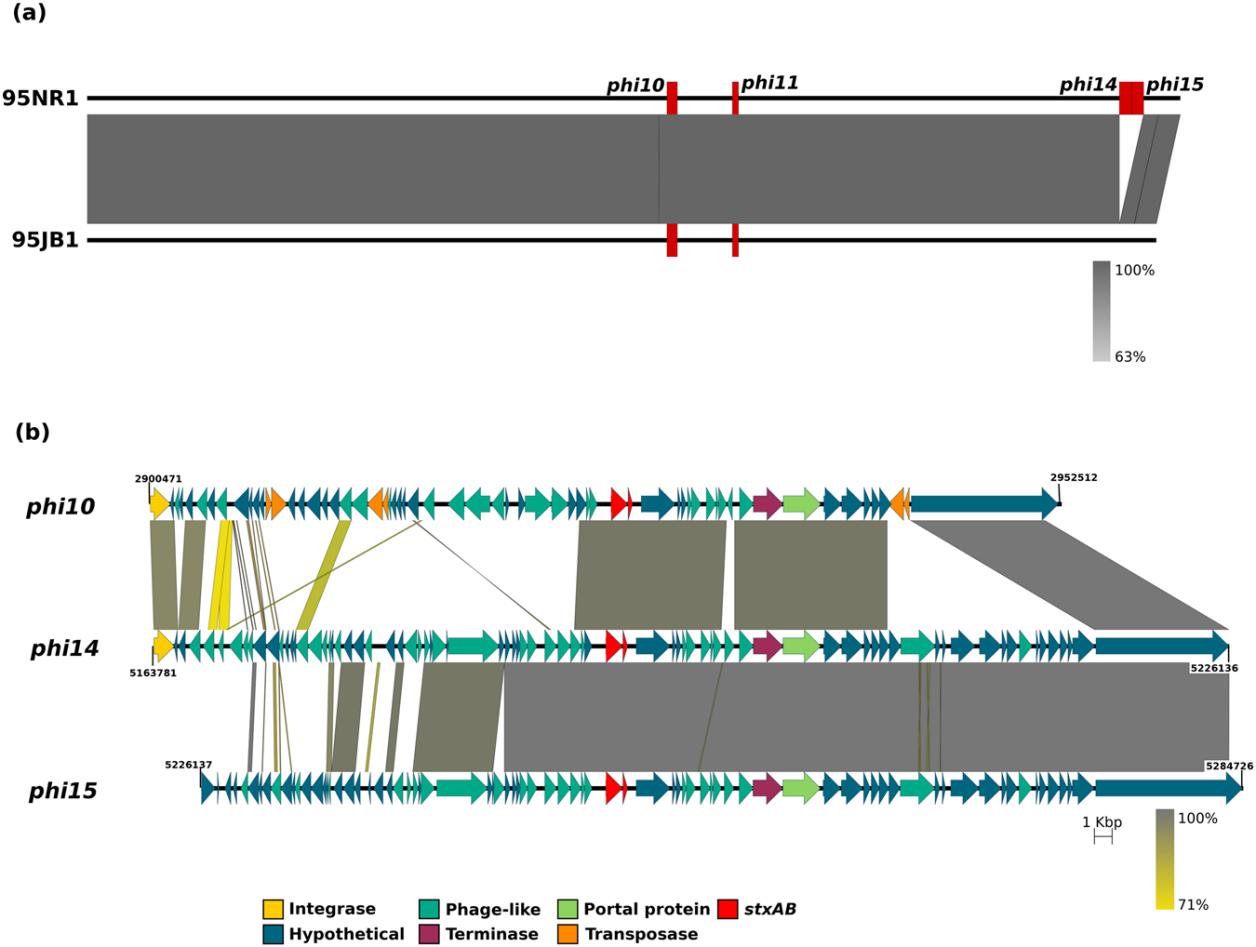
Comparison of *E. coli* 95JB1 and 95NR1 highlighting the position and context of Stx-carrying prophage. (**a**) Pairwise nucleotide comparison of 95NR1 (top) and 95JB1 (bottom) chromosomes. The chromosomes of 95NR1 and 95JB1 are represented to scale by the black bar with the Stx-carrying prophage insertion points indicated with red rectangles. Popouts display schematic representations of the four Stx-converting prophages carried by 95NR1. (**b**) Pairwise nucleotide comparison of three Stx2-converting prophages from 95NR1. Phi10, Phi14 and Phi15 are represented to scale. Prophage genes are represented by arrows coloured according to protein function as per the legend. Yellow and grey shading between phage represent regions of nucleotide sequence identity from 71% (yellow) to 100% (grey).

The ~120 kb difference in chromosome size between 95JB1 and 95NR1 was largely attributed to two tandemly inserted Stx2-converting prophages in 95NR1 (Phi14 and Phi15) that are absent from 95JB1 (Fig. 1a). Phi14 and Phi15 from 95NR1 are highly conserved and share 99% nucleotide sequence identity across 79% of their genomic sequence with regions of difference largely confined to the 5′ end of the prophage sequences (Fig. 1b). This pattern of sequence identity explains why assembly of this region was not possible using short read data alone. Indeed, the structure and context of all phage-encoding regions, including the Stx2 and Stx1 converting prophages (Phi10 and Phi11, respectively) carried by both outbreak strains (Table S1), were readily resolved in the complete PacBio assemblies.

### Insertion Sequence profiles differ between outbreak strains

To explore if there were any additional mobile genetic element differences between the outbreak strains we first compared the complete plasmids of each strain. Whereas the P1 plasmids were identical in both strains, the EHEC plasmid, p95JB1B, contained an additional IS*3* ssgr IS*51*-family insertion sequence (99% nucleotide sequence identity to IS*1203* from *E. coli* O111:H- PH) that was not present in p95NR1B (data not shown). This element (hereafter referred to as IS*1203*) has inserted into the 3′ end of the transposase EC95JB1_B00047 within an IS*91*-like insertion element.

**Figure 2:**
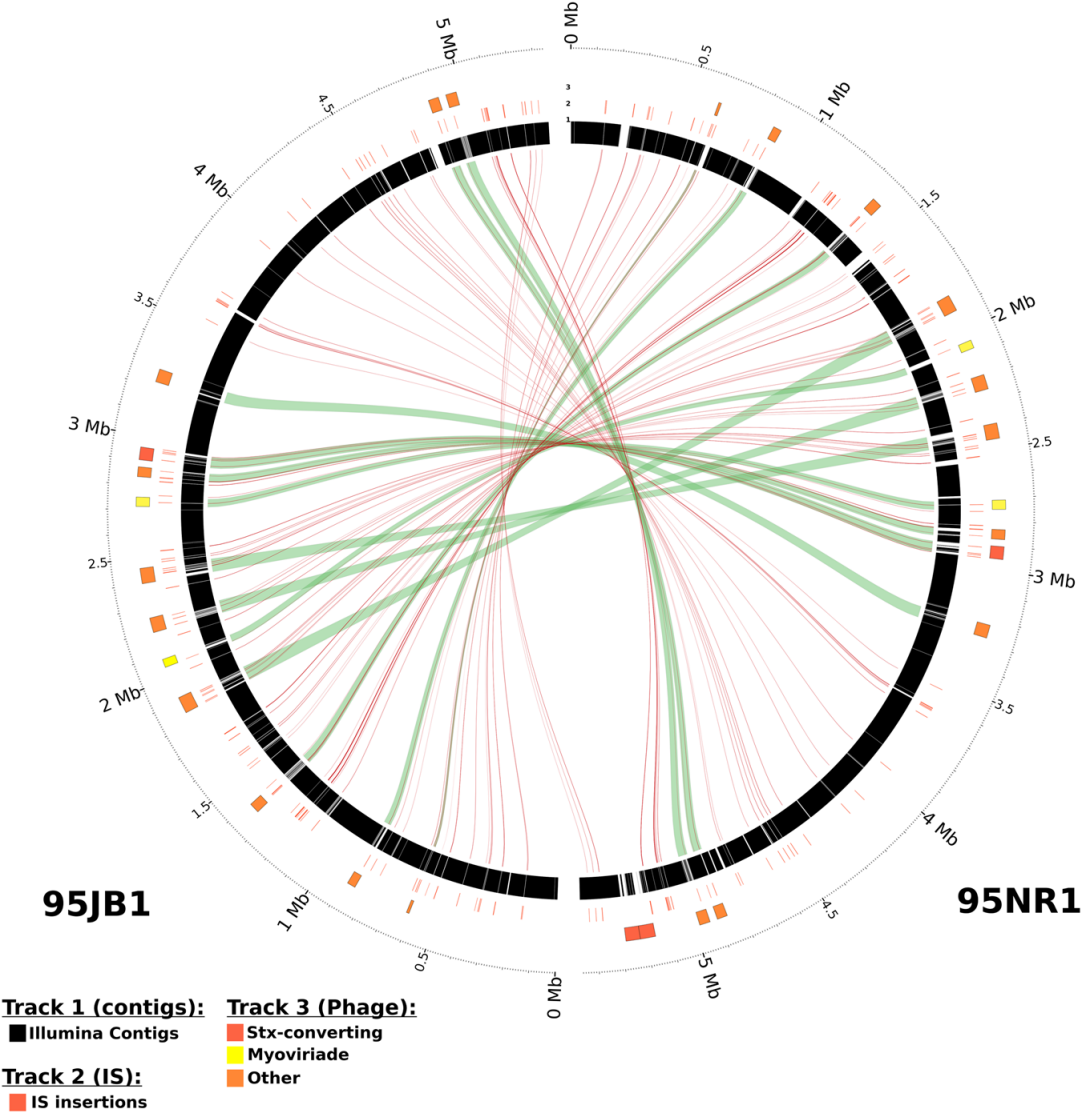
Comparison of 95JB1 and 95NR1 genome assemblies. Circos plot comparing prophage and insertion sequence (IS) content of 95JB1 (left) and 95NR1 (right). Putative prophage regions are highlighted on the outer most track by coloured rectangles: Stx-converting (Red), myoviriadae (yellow), other (orange). The position of all IS are represented by red bars on the middle track. Draft Illumina assemblies of 95JB1 and 95NR1, mapped to their complete genomes, are represented in black on the inner most ring where assembly gaps are shown as white space. Green ribbons connect prophage sequences common to both strains. Red lines connect IS that are common to both strains.

We next surveyed the chromosomal Insertion Sequence (IS) profiles of each outbreak strain (Fig. 2). Both 95JB1 and 95NR1 contain 17 different families of IS elements with two or more copies on their respective chromosomes (Table S2). Notably, 95JB1 encodes an additional chromosomal copy of an IS*3* ssgr IS*51*-family element (100% identical to the additional IS*1203* on p95JB1B) inserted at the 3′ end of EC95JB1_03899 (Table S3). EC95JB1_03899 is predicted to encode an ATPase. However, the IS has inserted ~150 bp upstream of the translational start site of a putative methyltransferase (MTase) gene (EC95JB1_03895), suggesting a possible functional role due to a polar effect on transcription.

### Three additional MTases are encoded on the additional 95NR1 Stx2 prophages

Bioinformatic characterisation of 95JB1 revealed that the strain encodes seven putative MTases in addition to Dam (target site: 5′-G^m6^ATC-3′) and Dcm (target site: 5′-C^m5^CWGG-3′). Five of these six putative MTases correspond to enzymes with known specificity that have been previously characterised in the O104:H4 serotype *E. coli* outbreak strain C227-ll^14^. These include homologs of the C227-11 orphan MTases M.EcoGI, M.EcoGII, M.EcoGVI and M.EcoGIX (M.Eco95JB1I, M.Eco95JB1II, M.Eco95JB1V and M.Eco95JB1VII, respectively), the Stx phage-borne M.EcoGIII (M.Eco95JB1III, encoded with its cognate restriction endonuclease (REase) on Stx2 prophage Phi10), and the Dam homolog M.EcoGV (M.Eco95JB1IV); the remaining MTase, Eco95JB1IX, is homologous to Type IIG bifunctional REase/MTase enzyme SenTFIV from *Salmonella enterica* subsp. enterica serovar Typhimurium (76% amino acid similarity) (Table 3).

**Table 3:** Summary of Methyltransferase genes from the chromosomes of 95JB1 and 95NR1.

MTase	Type^1^	95JB1 Enzyme name	95NR1 Enzyme name	Predicted Specificity^2^	Homolog^1^ (% aa similarity)
I	II	M.Eco95JB1I	M.Eco95NR1I	nonspecific	M.EcoGI/GII (92.8/92.4)
II	II	M.Eco95JB1II	M.Eco95NR1II	nonspecific	M.EcoGI/GII (93.6/93.1)
Dcm	II	M.Eco95JB1Dcm	M.Eco95NR1Dcm	CCWGG	M.EcoGDcm (98.5)
III	II	M.Eco95JB1III	M.Eco95NR1III	CTGCAG	M.EcoGIII (99.7)
IV	II	M.Eco95JB1IV	M.Eco95NR1IV	GATC	M.EcoGV (99.6)
V	II	M.Eco95JB1V	M.Eco95NR1V	ATGCAT	M.EcoGVI (98.6)
Dam	II	M.Eco95JB1Dam	M.Eco95NR1Dam	GATC	M.EcoGDam (99.7)
VI	II	—	M.Eco95NR1VI	GATC	M.EcoGV (99.4)
VII	II	—	M.Eco95NR1VII	GATC	M.EcoVT2 (99.2)
VIII	II	—	M.Eco95NR1VIII	GATC	M.EcoVT2 (99.2)
IX	IIG^4^	Eco95JB1IX	Eco95NR1IX	CRARCAG^3^	SenTFIV (76)
X	II	M.Eco95JB1X	M.Eco95NR1X	SAY	M.EcoGIX (96)

^1^Methyltransferases were classified based on similarity searches with the REBASE database^19^.

^2^Specificities only included if determined by PacBio.

^3^Predicted recognition motif based on the *in silico* bioinformatic characterisation of all MTase in 95JB1 and 95NR1 (this study).

^4^Type IIG enzymes are bifunctional with both REase and MTase capabilities^19^.

In addition to encoding all MTase-encoding genes identified in 95JB1, 95NR1 also encodes three additional Dam homologs encoded on its additional Stx2-converting prophages (Table 3). These comprise: (i) an additional copy of the Dam homolog M.EcoGV located on Stx2 prophage Phi14 (M.Eco95NR1VI), (ii) an identical copy of the orphan MTase M.EcoVT2Dam of *E. coli* prophage VT2-Sa on Stx2 prophage Phi14 (M.Eco95NR1VII) and (iii) a second identical copy of M.EcoVT2Dam on Stx2 prophage Phi15 (M.Eco95NR1VIII).

### The methylomes of 95JB1 and 95NR1 show remarkably different methylation patterns

Using the in-built capacity of PacBio to detect methylated nucleotides we detected two distinct recognition motifs (5′-G^m6^ATC-3′ and 5′-CTGC^m6^AG-3′) in 95JB1 and 95NR1, that match MTases with known specificities. 5′-GATC-3′ is a well characterised methylation motif, routinely identified in *E. coli* methylome analyses^14–17^ and known to be targeted by Dam^18^. We predict that the 5′-CTGCAG-3′ motif is targeted by MTases in 95JB1 and 95NR1 (M.Eco95JB1III/M.Eco95NR1III, respectively) that share 99% amino acid identity with M.EcoGIII, a previously characterised PstI-like Type II RM system shown to methylate and cleave 5′-CTGCAG-3′^14^. No additional methylated motifs in 95JB1 were detected suggesting that the remaining putative MTases are inactive under the tested conditions or could not be distinguished from each other (in the case of Dam and the Dam homologs) (Table 4). Dcm-mediated methylation of cytosine (5′-C^m5^CWGG-3′) could not be detected using the method used in this study.

**Table 4:** MTase recognition motifs identified in 95JB1 and 95NR1.

Motif	Modification Type	Number Detected	Number in Chromosome	Methylated (%)	Mean IPD Ratio
**95JB1**					
CTGCAG	m6A	2390	2390	100.0	6.9934053
GATC	m6A	41658	41686	99.9	5.6757846
**95NR1**					
CTGCAG	m6A	2434	2434	100.0	7.1674423
CRARCAG	m6A	4074	4074	100.0	7.1986117
GATC	m6A	42242	42270	99.9	5.794127

Remarkably, 95NR1 also contained more than 4000 methylated bases corresponding to a third motif, 5′-CRARC^m6^AG-3′ (Table 4). As no methylation was detected at the corresponding motifs in 95JB1 we considered that this new activity must relate to an MTase that is not present or non-functional in 95JB1. Screening the 5′-CRARCAG-3′ motif against REBASE^19^ confirmed that a cognate MTase enzyme has not been previously characterised and to the best of our knowledge it represents a novel MTase target recognition site (Table 4).

### A novel MTase responsible for methylation of the CRARCAG motif

In order to identify the MTase responsible for methylation of the 5′-CRARCAG-3′ motif in 95NR1 (but not 95JB1) we first examined the specificities of the experimentally determined *E. coli* C227-11 MTases for which there are five close homologs in 95JB1/95NR1 (>92% amino acid sequence identity; Table 3)^14^. Based on the high amino acid identity between these homologs it is highly unlikely that any C227-11 MTase homologs could be responsible for methylation of the 5′-CRARCAG-3′ motif. Similarly, M.EcoVT2Dam is known to target 5′-GATC-3′; thus both homologs of M.EcoVT2Dam in 95NR1 (99% amino acid identity) would also be expected to target 5′-GATC-3′^20^. The remaining candidate MTase encoded by Eco95NR1IX shares 76% amino acid identity to SenTFIV, a Type IIG R-M bifunctional enzyme which targets the GATCAG recognition site. Minor difference in the target recognition domain (TRD) of homologous MTase enzymes can result in major differences in target specificity as exemplified in *E. coli* EC958 where the Type IIG R-M system EcoMVII and SenTFIV share 68% amino acid identity, but their target sites are very different (5′-CANCATC-3′ and 5′-GATCAG-3′, respectively). Furthermore, 100% hemi-methylation of the 5′-CRARCAG-3′ motif, as observed in 95NR1, is characteristic of the Type IIG family of MTases. Based on these observations, we propose that Eco95NR1IX catalyses CRARCAG modification in 95NR1 and that the lack of corresponding methylation in 95JB1 is due to IS-mediated transcriptional inactivation of the orthologous Eco95JB1VI gene.

### IS insertion is predicted to prevent transcription of the M.Eco95JB1IX gene

BLAST comparison (blastp, E-value cutoff = 1e^−3^) of Eco95NR1IX against a database of complete *E. coli* genomes identified homologs in four additional *E. coli* strains: *E. coli* str. HS (EcHS_A0339; 76% amino acid sequence identity), *E. coli* O111:H- str. 11128 (ECO111_5156; 100% amino acid sequence identity), *E. coli* str. ‘clone i2’ (i02_4877; 76% amino acid sequence identity) and *E. coli* str. ‘clone i14’ (i14_4877; 76% amino acid sequence identity). With the exception of ECO111_5156, homologs of Eco95NR1IX exhibited extensive variation in their TRDs and are likely to have different target specificities (Fig. S1a). Interestingly, genes upstream of *eco95NR*1*IX* are highly conserved in all strains (Fig. S1b) and suggest that *eco95NR1IX* could be transcribed as part of a large 8.5 kb operon encoding a Type IIG REase/MTase enzyme (Eco95NR1IX), a putative ATPase (EC95NR1_04072) and two hypothetical proteins whose function is currently unknown (EC95NR1_04073 and EC95NR1_04074) (Fig. S2; Table S4). A putative promoter region (5′-TGGCAT-14 bp-CATTACAAT-3′) 50 bp upstream of EC95NR1_04074 could indicate the primary transcriptional start site for this operon. Predicted promoter regions were also identified 68 bp upstream of EC95NR1_04072 and overlapping the predicted start codon of *eco95NR1IX*. An additional predicted promoter region was observed in 95JB1 which was located on the boundary of the IS insertion into EC95JB1_03895 (Fig. S2). Based on these observations we propose that the IS insertion into EC95JB1_03899 results in premature transcriptional termination of the operon leaving *eco95JB1IX* untranscribed.

### Potential functional consequences of CRARCAG methylation

To determine if methylation of 5′-CRARCAG-3′ motifs could have a functional role in the genome of 95NR1 we analysed the distribution of sites located within 300 bp upstream of an annotated start codon. We identified 871 candidate genes where 5′-CRARCAG-3′ methylation might have a regulatory role. These include the flagellar genes *fliCDSHIJKQPR*, *flgCJG* and the flagellar regulator *flk*; Type II secretion associated genes *gspMLJHGE*; Shiga toxin gene *stx*_*1*_*B* (Phi11); the two component regulatory system BaeSR, and EutR, a transcriptional regulator associated with EHEC pathogenesis^21^. Upstream sequences were further analysed for the presence of putative promoter regions using Neural Network Promoter Prediction^22,23^. A total of 601 of the 871 candidate genes were found to contain putative promoter regions within 300 bp of their start codon, 177 of which contained a methylated 5′-CRARCAG-3′ motif (Table S5). Clustering of these genes based on the functional class of their encoded proteins revealed no significant enrichment in any functional category when compared to the functional clustering of all genes (Fig. S3).

### Distribution of Methylated motifs reveals difference in methylation patterns within prophage sequences

**Figure 3:**
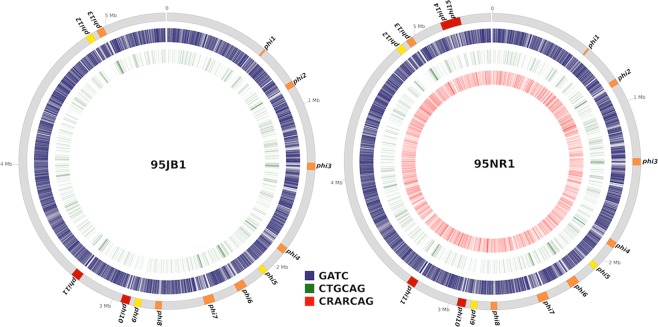
Comparison of methylated DNA on the chromosomes of 95JB1 and 95NR1. Circos plot displaying the distribution of methylated nucleotides on the chromosomes of *E. coli* 95JB1 (left) and 95NR1 (right). Prophage insertion points are highlighted on the outer- most track in orange with Stx-carrying prophage in red and myoviriadae in yellow. The remaining coloured tracks highlight the chromosomal positions of all methylated sites for each motif. Tracks are coloured as per the legend; GATC, red; CTGCAG, green; CRARCAG, red.

There was an observed difference in the distribution of the 5′-GATC-3′ motif between the core genome and the prophages in both 95NR1 and 95JB1 (Fig. 3). This difference is attributable to the presence of GATC-free regions within the prophages and is suggestive of selection against Dam methylation in certain phage genes. Differences in the distribution of GATC sites between the core and accessory genome have previously been described in *E. coli* K12^24^ and more recently in *E. coli* EC958^16^. Interestingly, these prophage-associated GATC-free regions are enriched for the 5′-CTGCAG-3′ motif found in most prophages in 95JB1 and 95NR1, with the exception of Phi9 (unclassified Myoviridae) and Phi12 (Mu-like Myoviridae) where the 5′-CTGCAG-3′ motif is entirely absent making it significantly under-represented compared to the core genome (P ≤ 0.0001). In 95NR1 the 5′-CRARCAG-3′ motif appears to be randomly distributed throughout the genome and exhibited no significant enrichment bias to either the core or accessory genome.

## Discussion

*E. coli* 95JB1 and 95NR1 are two isolates from a historical HUS outbreak in Adelaide, Australia in 1995^1,3^. 95NR1, the more virulent of the two strains, is characterised by the presence of two additional Stx2 prophages and two additional copies of *stx*_2_*AB* when compared to 95JB1^4^. Previous analysis of the Illumina draft genomes of these outbreak strains showed that, relative to 95NR1, 95JB1 had acquired a handful of SNPs and had lost two Stx2 prophages. Despite these findings it was not possible to resolve the sequences of most mobile genetic elements (including prophages) due to the fragmented nature of the draft assembly. In this study we hypothesised that there were other genetic differences between the strains that may account for the different virulence profiles. Using SMRT sequencing to produce complete genomes of these important historical isolates enabled the complete definition of the structure and context of all prophages (including the tandemly arranged Stx prophages Phi 14 and Phi15 in 95NR1). Our comparison of the complete genomes of 95JB1 and 95NR1 did not identify any additional differences in known virulence genes, beyond the previously described differences in *stx2AB* gene copy number^4^. However, by identifying the position and context of all IS elements in both genomes we identified an additional chromosomal copy of an IS*1203* element in 95JB1 that likely prevents transcription of a Type IIG REase/MTase encoded nearby. By analysing the kinetic variation data produced during sequencing on the SMRT platform we found a difference between the methylomes of 95JB1 and 95NR1. As we are able to exclude all other MTases encoded by 95NR1 we conclude that this IS difference accounts for the different methylation patterns between the strains. It has been well established that DNA methylation can play an active role in virulence and gene regulation^25–28^. By examining bases methylated within promoter regions in 95NR1, but not 95JB1, we have identified a number of potential differentially regulated genes that could contribute, directly or indirectly, to the increased virulence observed in 95NR1. In light of our findings a comparison of gene expression between 95JB1 and 95NR1 provides an intriguing avenue for future research.

Previously, a detailed analysis of the Illumina draft genome assemblies of 95JB1 and 95NR1 enabled accurate determination of prophage insertion sites, plasmid content and the multiplicity of *stx* genes^4^. Only one of the phage integration sites predicted by Illumina read-mapping alone was a false positive (tRNA-Arg), due to an IS*1203* insertion. As expected, repeat elements were the major cause of fragmentation in the draft genomes of 95JB1 and 95NR1, with the majority of contigs terminating at IS elements or within prophage regions. Long SMRT sequencing reads easily bridged these repeat regions generating complete, high quality assemblies with little manual intervention. Complete genomes are a vital resource when characterising the genetic variation which exists between strains, as the positions of IS and other mobile elements can influence host biological processes, including pathogenicity and antibiotic resistance^29–33^.

Surprisingly, we found that the most likely explanation for the observed differences in methylation between the outbreak strains was the insertion of an additional IS*1203* upstream of the gene encoding M.Eco95JB1IX in 95JB1. This particular IS element was first identified in *E. coli* O111:H- strain PH^34^ and has previously been associated with insertional inactivation of genes in STEC^35–37^. Although formal demonstration that the 5′-CRARCAG-3′ recognition motif is methylated by Eco95NR1IX would require methylome profiling of a 95NR1 Eco95NR1IX deletion mutant, several lines of evidence support our contention that Eco95NR1IX/Eco95JB1IX catalyses the methylation of the 5′-CRARC^m6^AG-3′ motif. Other than Eco95NR1IX there were no candidate MTases in the 95NR1 or 95JB1 genomes that could account for the methylation of the 5′-CRARCAG-3′ motif in 95NR1 alone. Eco95NR1IX and SenTFIV share 76% amino acid identity consistent with a different recognition site. Furthermore, the distinctive pattern of hemi-methylation of the 5′-CRARCAG-3′ motif, as observed in 95NR1, is characteristic of the Type IIG family of MTases. We also note that since preparation of our manuscript this motif has been linked with Eco95NR1IX homologs in other PacBio *E. coli* genomes in the REBASE database^19^.

We identified two active MTases common to both 95JB1 and 95NR1: Dam and the recently characterised Stx phage-encoded RM system methyltransferase M.EcoGIII, which recognises the CTGCAG motif. M.EcoGIII is known to affect the expression of 1,951 genes in *E.coli* C227-ll, provides resistance to infection by other lambda-like phage and influences growth^14^. It is likely that M.EcoGIII occupies a similar regulatory role in 95JB1 and 95NR1 as it does in C227-11. However, determining the functional consequences of CTGCAG methylation in 95JB1 and 95NR1 requires further analysis. Interestingly, three prophage loci in C227-11 were found to be enriched for the CTGCAG motif^14^ and a similar enrichment of the CTGCAG motif was also observed in most prophages of 95JB1 and 95NR1. In contrast, two prophage regions in 95JB1 and 95NR1 (Phi9 and Phi12) contained no CTGCAG motifs, suggesting selection against the presence of these sites. In C227-11 the EcoGIII RM system was shown to protect against infection by other lambda-like phages, but T4 phages were resistant to restriction (despite containing CTGCAG sites), which could be attributed to heavy modification of T4 phage genomes^14^. Phi9 and Phi12 are lysogenic members of the Myoviridae, the same family of virus to which the lytic T4 phage belong, but substantially different at the genome level. The lack of any CTGCAG motifs in Phi9 and Phi12 (CTGCAG motif is expected to occur by chance every 4064 bp) suggests active selection against this motif, which would presumably render the Phi9 and Phi12 genomes immune to digestion by R.EcoGIII.

Bacterial MTases are known to have diverse roles which include regulation of gene expression. For example, the orphan MTase Dam has been established as regulator of gene expression in other *E. coli* species and recently the Type II RM system EcoGIII was shown to directly or indirectly affect the expression of ~1900 *E. coli* genes^14,25,27^. Similarly, it has been suggested that Type IIG RM systems might have a role in bacterial genomes other than protection^38^. The proximity of methylated 5′-CRARCAG-3′ sites to the predicted promotor regions of 177 genes in 95NR1 suggests that methylation of this motif could directly cause differential gene expression between 95NR1 and 95JB1. We have identified several operons and regulators that may be likely candidates for differential expression, although it should be noted that putative promoter sites were predicted *in silico* and may not reflect true promotor regions. It is also worth noting that 95NR1 and 95JB1 have been classified as non-motile (H-) according to their original serotyping results suggesting that differential expression of flagellar loci is unlikely to result in phenotypic difference^3^. Analysis of the transcriptome using RNA-Seq will be necessary to fully define the influence of methylation on differential regulation between these otherwise very similar strains.

DNA modifications are an established cause of phenotypic heterogeneity in both isogenic and clonal populations^39^. Phase variation or ON/OFF switching of the pyelonephritis-associated pili (Pap) operon and antigen 43 (Ag43) are two of the most well characterised examples of methylation-mediated intercellular heterogeneity in *E. coli*^40,41^. In these examples, differences in the methylation status of GATC motifs (mediated by Dam) in the promoter regions of *pap* and *ag43* control ON/OFF expression of these loci and intercellular heterogeneity within the clonal population. Differences in MTase activity in 95NR1 and 95JB1 (due to Eco95NR1IX and Eco95JB1IX, respectively) is a clear example of epigenetic heterogeneity which has arisen within this clonal population. Whether this observed epigenetic heterogeneity is driving phenotypic difference within the population is currently unknown and represents an interesting avenue for further research.

During our characterisation of the 95NR1 and 95JB1 methylomes we made the surprising discovery that a Type IIG MTase carried by 95NR1 and 95JB1 is likely encoded as part of an operon. To the best of our knowledge a Type IIG MTase has not previously been reported as a component of a multi-gene system. The function of the other proteins in this putative operon and whether they are linked to the activity of the MTase is currently not known. Despite identifying a putative primary promoter at the 5′ operon boundary, the presence of several additional promoters distributed through the operon raises the possibility that some genes are transcribed separately. Future analysis of the transcriptome of 95NR1 or other *E. coli* that carry homologs of M.Eco95NR1IX will be necessary to correctly determine transcriptional start-sites. Notably, SMRT sequencing also offers the potential for characterising complete polycistronic mRNAs by adapting the Iso-Seq protocol^42,43^.

Characterising the genetic differences between strains is highly important for determining the evolutionary history of bacterial populations, tracking clinical outbreaks and identifying functional mutations which contribute to virulence and antibiotic resistance^44–47^. Currently, Illumina is the platform of choice for studying single nucleotide variation, due to its capacity for accurate high throughput sequencing of hundreds or thousands of strains. In this study the SNP profile between 95JB1 and 95NR1 was identical using PacBio or Illumina data, whereas only PacBio could accurately resolve the mobile genetic element content of both strains. Of particular importance to our understanding of STEC biology was the complete resolution of two tandemly arrayed Stx2 prophages encoded by 95NR1 and not 95JB1. The complete sequence of three full-length Stx2 prophages was determined in 95NR1 highlighting the difficulty of resolving multiple similar prophage genomes within a draft assembly. The *stx*2 genes of Phi14 and Phi15 were both on 4.83 kb *Eco*RI restriction fragments which explains why only one additional *stx*2-specific band was identified by Southern hybridization in the original report^1^. Although a highly accurate WGS method for subtyping *stx* genes has been developed^48^, the reliance of this method on short read sequencing data means it lacks the discriminatory power to determine whether multiple copies of the same subtype are due to multiple insertions by different Stx-converting phage or as a result of gene duplication. Our study highlights how population genomic studies of STEC outbreaks or global collections could benefit from SMRT sequencing and/or bioinformatics approaches that take into account mobile genetic element heterogeneity.

Although the ability of SMRT sequencing to resolve large mobile genetic elements is well documented^29–33^, it is important to recognise that small plasmids are easily lost during the library preparation for PacBio. On the PacBio instruments the size distribution of the SMRTBell sequencing libraries influences read length performance. For example, short DNA library fragments preferentially load into the sequencing wells (Zero-mode wave guides) on the SMRT Cell limiting the long read potential of the sequencing library. In order to maximise the lengths of reads prior to sequencing it is necessary to filter small fragments from the library and narrow its size distribution. In this study targeted DNA size selection was achieved using the BluePippin instrument (http://www.sagescience.com). Using BluePippin small DNA fragments are separated from larger fragments enabling the collection of sequencing libraries with narrower size distributions. Both 95NR1 and 95JB1 were sequenced using BluePippin size-selected 20 kb SMRTBell libraries. As small plasmid DNA was visible in the original DNA extraction (data not shown) it appears that DNA fragments representing the missing colicin plasmids of 95NR1 and 95JB1 were filtered from the sequencing library during targeted size selection.

In conclusion, our study reveals that in addition to acquiring a small number of SNPs and losing two tandemly arranged Stx2 prophages, 95JB1 has also lost the activity of a novel MTase (apparently via IS insertion), that may influence the transcription of several hundred genes. PacBio SMRT sequencing has enormous potential to reveal the genetic and epigenetic heterogeneity within a clonal population. Further genomic analysis of IS and prophages within closely related STEC strains will further build our understanding of short-term evolution and strain heterogeneity within the context of an outbreak.

## Methods

### Genome sequencing and assembly

Genomic DNA (gDNA) from *E. coli* strains 95NR1 and 95JB1 was sequenced on a PacBio RSII instrument (University of Queensland Centre for Clinical Genomics; UQCCG) using two SMRT cells, a 20 kb insert library and the P6 polymerase and C4 sequencing chemistry. *De novo* assembly of the raw PacBio sequencing data was done using the hierarchical genome assembly process (HGAP version 2) and Quiver^9^ from the SMRT Analysis software suite (version 2.3.0 – http://www.pacb.com/devnet/) with default parameters. Following *de novo* assembly all contigs were visually screened for overlapping sequences on their 5′ and 3′ ends using contiguity (https://github.com/mjsull/Contiguity)^49^. Overlapping ends, a characteristic feature of the HGAP assembly process, were manually trimmed based on sequence similarity and the contigs were circularised. Circularised contigs (chromosome and plasmids) were then subjected to a polishing phase, were the raw PacBio sequencing reads were mapped back onto the assembled circular contigs (BLASR^50^ and quiver) to validate the assembly and resolve any remaining errors. Following multiple rounds of polishing an additional improvement step was required to resolve single nucleotide insertion and deletion errors associated with homopolymer tracts. Reads from the publically available Illumina sequence data for 95NR1 (SRA accession: SRR953500) and 95JB1 (SRA accession: SRR954273) were aligned to their respective genomes using bwa version: 0.7.12-r1039^51^ and a corrected consensus was called using Pilon version 1.18^52^. A total of 42 indels were corrected in 95NR1 and 30 indels in 95JB1. 95NR1 was further processed as the incorrect distribution of reads between two tandemly, integrated Stx prophages initially resulted in a misassembly characterised by a contig break. This misassembly was manually corrected, and verified by realigning the raw reads to the complete chromosome.

### Methylome analysis

The detection of methylated bases and clustering of modified sites to identify methylation-associated motifs was performed using the RS_Modification_and_Motif_analysis.1 tool from the SMRT analysis package version 2.3.0. Raw reads were aligned to the complete genomes of 95JB1 and 95NR1 and interpulse duration (IPD) ratios were calculated using PacBio’s *in silico* kinetic reference computational model (http://www.pacb.com/wp-content/uploads/2015/09/WP_Detecting_DNA_Base_Modifications_Using_SMRT_Sequencing.pdf).

To compare the CTGCAG motif distribution of Phi9 and Phi12 with the rest of the chromosome, the sequence for each strand was split into 1000 bp segments with a 250 bp overlap using Bedtools v2.17.0^53^. Analysis of the mean distribution of CTGCAG motifs per segment within these genomic regions was performed as previously described using a custom analysis of variance (ANOVA) R script, which adjusts for heteroscedasticity^16^.

### SNP analysis of 95NR1 and 95JB1

To determine the number and position of unique SNPs that differentiate 95JB1 and 95NR1, Illumina reads were simulated from the complete genomes of 95NR1 and 95JB1 and aligned to the genome of *E. coli* O111:H- strain 11128. SNP calling and Indel prediction was performed using Nesoni and the Nesoni n-way pairwise comparison method was used to identify SNPs conserved in all three strains (http://www.vicbioinformatics.com/software.nesoni.shtml). Additionally, a reference free SNP analysis was performed by direct comparison of the complete genome of 95JB1 and 95NR1 using MUMmer version 3.2.3^54^.

### Genome annotation and comparative genomics

Initial gene calling and automated functional annotation of 95NR1 and 95JB1 were performed using Prokka (Prokka: Prokaryotic Genome Annotation System – http://vicbioinformatics.com/)^55^ using a custom *Escherichia* genus database consisting of protein sequences from the EcoCyc website (http://ecocyc.org/). Putative phage encoding loci and IS elements were identified using PHAST^56^ and ISfinder^57^, respectively, followed by manual curation of mobile element boundaries. PHAST predictions that were “questionable” or “incomplete” were not annotated, with the exception of those matching syntenic prophage regions in STEC O111 strain 11128 (Phi4 and Phi8) and *stx-*encoding prophages (Phi15). Artemis Comparison Tool (ACT)^58^, EasyFig^59^ and Circos^60^ were used to visually compare the genomes and methylomes of 95NR1 and 95JB1 and identify regions of similarity and difference. Methyltransferase genes and restriction modification systems were identified and annotated by comparison (BLASTn ≥95% nucleotide identity) of all coding sequences from 95JB1 and 95NR1 against the REBASE database^19^. Promoter sequences of the regions encoding *eco95NR1IX* and *eco95JB1IX* were predicted using BPROM (http://www.softberry.com/berry.phtml)^61^ with default settings.

### The functional characterisation of CRARCAG methylation

Genome-wide *in silico* prediction of promoter sequences was done using Neural Network Promoter Prediction version 2.2^22,23^ and KEGG gene ontologies (KOs) were assigned using BlastKOALA^62^. The proximity of the CRARCAG motifs to all protein coding regions was determined using custom Python scripts. Protein coding regions that did not contain a methylated CRARCAG motif within 300 bp of its start codon were excluded from the analysis. For the remaining protein coding regions, 300 bp of sequence upstream of their respective start codons was screened for putative promoter regions. CRARCAG motifs located within predicated promotor regions where identified, and KOs were assigned using BlastKOALA.

### Accession numbers

Genome data for *E. coli* 95JB1 and 95NR1 has been deposited to NCBI under Bioproject PRJNA383943 and PRJNA383942, respectively. Raw PacBio sequence read data for 95JB1 and 95NR1 has been deposited to the Sequence Read Archive under the accessions SRR5520357 and SRR5518882, respectively. The complete, annotated genome of 95JB1 has been deposited to Genbank (accession: CP021335-CP021337). The complete, annotated genome of 95NR1 has been deposited to Genbank (accession: CP021339-CP021341).

## Supplementary information

Supplementary Appendix

Supplementary Table S5
