# Supplementary material for: SMRT sequencing reveals differential patterns of methylation in two O111:H- STEC isolates from a hemolytic uremic syndrome outbreak in Australia
Source: Sci Rep. 2019 Jul 1;9:9436. doi: 10.1038/s41598-019-45760-5 (PMC6602927; doi:10.1038/s41598-019-45760-5)
Supplement: Supplementary file 1 — Supplementary Appendix [file 41598_2019_45760_MOESM1_ESM.pdf]

SMRT sequencing reveals differential patterns of methylation in two O111:H- STEC isolates from a hemolytic uremic syndrome outbreak in Australia

Brian M. Forde[1,2,4], Lauren J. McAllister[3], James C. Paton[3], Adrienne W. Paton[3], Scott A. Beatson[1,2,4*]

[1] Australian Infectious Diseases Centre, The University of Queensland, Brisbane, Australia [2]The School of Chemistry and Molecular Biosciences, The University of Queensland, Brisbane, Australia. [3]Research Centre for Infectious Diseases, Department of Molecular and Cellular Biology, University of Adelaide, Adelaide, SA 5005, Australia. [4]Australian Centre of Ecogenomics, The School of Chemistry and Molecular Biosciences, The University of Queensland, Australia.

Corresponding author:

* Scott A. Beatson, School of Chemistry and Molecular Biosciences, The University of Queensland, Brisbane 4072, QLD, Australia; Telephone +61-7-33654863; email s.beatson@uq.edu.au

Supplementary Tables and Figures

Table S1: Prophage regions identified in 95JB1 and 95NR1

Phage	Type	95JB1	95NR1	Position 95JB1 (start..stop)	Postion 95NR1 (start..stop)
Phi1	Lambda-like	+	+	608798..620241	608798..620241
Phi2	Lambda-like	+	+	851546..885955	851546..885954
Phi3	Lambda-like	+	+	1354451..1388510	1354451..1388510
Phi4	Lambda-like	+	+	1854751..1907596	1855102..1907920
Phi5	Unclassified Myoviridae	+	+	2048061..2079037	2048393..2079387
Phi6	Lambda-like	+	+	2200935..2232970	2201281..2233320
Phi7	Lambda-like	+	+	2399479..2446370	2399829..2446720
Phi8	Lambda-like	+	+	2713825..2734495	2714175..2734845
Phi9	Unclassified Myoviridae	+	+	2832770..2871338	2833098..2871688
Phi10	Lambda-like Stx2-converting	+	+	2900178..2952166	2900528..2952516
Phi11	Lambda-like Stx1-converting	+	+	3227785..3257795	3228135..3258147
Phi12	Mu-like Myoviridae	+	+	4862178..4899383	4862525..4899734
Phi13	Lambda-like	+	+	4935870..4979657	4936221..4980008
Phi14	Lambda-like Stx2-converting	-	+	NA	5164024..5226171
Phi15	Lambda-like Stx2-converting	-	+	NA	5226172..5284858

Table S2: Chromosomal IS elements of 95JB1 and 95NR1

Family	Count[1]
IS1	4/4
IS110	2/2
IS200/IS605	4/4
IS256	3/3
IS3 ssgr IS2	4/4
IS3 ssgr IS3	8/8
IS3 ssgr IS51 (IS*1203*)	33/32[2]
IS30	2/2
IS4	1/1
IS4 ssgr IS50	2/2
IS481	1/1
IS630	3/3
IS66	5/5
IS91	8/8
ISAs1	6/6

ISL3	1/1
ISNCY ssgr ISPlu15	5/5

[1] Number of each IS family in 95JB1 and 95NR1 (95JB1/95NR1)

[2] 95JB1 contains an additional IS3 ssgr IS51 family element (99% identical to IS*1203* from *E. coli* O111:H- PH).

Table S3: Context of IS elements identified in 95JB1 and 95NR1

		95JB1		95NR1	
**family**	**ori**	**Start**	**Stop**	**Start**	**Stop**
ISNCY ssgr ISPlu15	+	148395	149354	148452	149354
IS3 ssgr IS51 (IS*1203*)	+	151744	153019	151798	153011
ISAs1	+	270948	272204	271064	272187
ISAs1	+	272386	273429	272386	273429
IS3 ssgr IS51 (IS*1203*)	+	335029	336245	335032	336245
IS3 ssgr IS51 (IS*1203*)	+	339373	340586	339376	340589
Tn3	-	354228	353635	354231	353638
IS3 ssgr IS51 (IS*1203*)	+	432718	433931	432719	433932
IS3 ssgr IS3	+	533291	534468	533292	534469
IS110	-	535334	534438	535335	534439
IS481	-	574130	573759	574130	573759
IS3 ssgr IS3	+	611904	613081	611904	613081
IS3 ssgr IS51 (IS*1203*)	+	617925	619138	617924	619137
ISAs1	-	631995	630859	631994	630858
ISAs1	+	782753	783421	782753	783421
Tn3	-	828885	828007	828885	828007
IS3 ssgr IS51 (IS*1203*)	+	861294	862507	861294	862507
IS3 ssgr IS51 (IS*1203*)	-	1133448	1132235	1133447	1132234
IS1	+	1194339	1195024	1194338	1195023
IS30	-	1202001	1202243	1202241	1201999
IS66	-	1204615	1202282	1204613	1202280
IS30	-	1204670	1204981	1204979	1204677
IS3 ssgr IS3	+	1205043	1205944	1205041	1205942
IS3 ssgr IS3	-	1216337	1215996	1216335	1215994
IS66	-	1221114	1220734	1221217	1220732
IS66	+	1221602	1223954	1221600	1223952
IS66	-	1225554	1223958	1225552	1223956
IS1	-	1248705	1248056	1248703	1248054
IS3 ssgr IS3	+	1355913	1357090	1355910	1357087
IS3 ssgr IS51	-	1361120	1359907	1361117	1359904
IS3 ssgr IS51	-	1363026	1361813	1363023	1361810
IS256	+	1395226	1395804	1395211	1395801
IS4	-	1505593	1504940	1505639	1504987
Tn3	+	1520732	1521280	1520778	1521326
IS3 ssgr IS51	-	1576314	1575101	1576663	1575450
IS3 ssgr IS3	-	1586625	1585448	1586625	1585796
ISAs1	-	1654538	1653402	1654886	1653750
IS1	+	1654905	1655021	1655253	1655369
IS200/IS605	-	1687469	1686261	1687817	1686609
IS200/IS605	+	1687498	1687900	1687846	1688248
IS3 ssgr IS51 (IS*1203*)	+	1688394	1689607	1688742	1689955
IS1	-	1846488	1845840	1846837	1846189
IS3 ssgr IS51 (IS*1203*)	+	1850122	1851335	1850471	1851684

IS630	+	1875165	1876006	1875513	1876354
IS256	+	1879388	1879971	1879736	1880319
IS91	+	1893006	1894238	1893354	1894586
IS3 ssgr IS51 (IS*1203*)	+	1899397	1900610	1899745	1900958
IS3 ssgr IS51 (IS*1203*)	-	2003959	2002746	2004308	2003095
IS91	-	2048628	2048047	2048977	2048396
IS200/IS605	-	2155768	2154560	2156117	2154909
IS200/IS605	+	2155794	2156204	2156143	2156553
IS3 ssgr IS51 (IS*1203*)	-	2202148	2200935	2202497	2201284
IS3 ssgr IS51 (IS*1203*)	+	2226665	2227878	2227013	2228226
IS21	-	2244628	2243888	2244976	2244236
IS200/IS605	-	2360741	2359533	2361089	2359881
IS200/IS605	+	2360764	2361177	2361112	2361525
IS66	+	2425925	2426287	2426273	2426635
IS66	+	2426338	2426634	2426686	2426982
IS66	+	2426665	2428278	2427013	2428626
IS21	-	2436554	2435808	2436902	2436156
IS3 ssgr IS51 (IS*1203*)	-	2445392	2444179	2445739	2444526
IS3 ssgr IS51 (IS*1203*)	-	2476044	2474882	2476391	2475229
IS3 ssgr IS51 (IS*1203*)	-	2486065	2484852	2486412	2485199
IS3 ssgr IS2	-	2522346	2521118	2522693	2521465
IS30	-	2523355	2522435	2523702	2522782
IS3 ssgr IS3	-	2523680	2523312	2524027	2523659
IS3 ssgr IS51 (IS*1203*)	+	2727214	2728427	2727560	2728773
ISNCY ssgr ISPlu15	+	2756883	2757785	2757229	2758131
ISNCY ssgr ISPlu15	-	2816949	2816059	2817295	2816405
IS3 ssgr IS51 (IS*1203*)	-	2818246	2817033	2818592	2817379
IS91	-	2833758	2832754	2834104	2833100
IS3 ssgr IS51 (IS*1203*)	-	2835645	2834432	2835991	2834778
Tn3	+	2861718	2862317	2862064	2862663
IS3 ssgr IS51 (IS*1203*)	+	2908658	2909871	2909005	2910218
IS3 ssgr IS51 (IS*1203*)	+	2938372	2939585	2938719	2939932
IS3 ssgr IS51 (IS*1203*)	-	2945355	2944142	2945702	2944489
IS3 ssgr IS51 (IS*1203*)	+	2952982	2954195	2953329	2954542
IS91	-	3543635	3542739	3543984	3543088
IS66	-	3630986	3630735	3631335	3631084
IS3 ssgr IS51 (IS*1203*)	+	3631062	3632275	3631411	3632624
IS4 ssgr IS50	+	3632635	3633525	3632984	3633874
IS1	+	3636182	3636328	3636531	3636677
IS630	+	3638662	3639573	3639011	3639922
IS3 ssgr IS2	+	3639734	3640057	3640083	3640406
IS3 ssgr IS3	-	3643941	3642779	3644290	3643128
IS3 ssgr IS2	-	3677398	3676206	3677748	3676556
ISL3	+	3949734	3950781	3950082	3951129
ISNCY ssgr ISPlu15	+	4118915	4119793	4119261	4120139
ISAs1	+	4202490	4203626	4202836	4203972
IS3 ssgr IS2	+	4416923	4418151	4417270	4418498
IS21	-	4486278	4484875	4486625	4485222
IS3 ssgr IS51 (IS*1203*)	-	4502380	4501167	4502727	4501514
IS91	+	4524870	4526102	4525217	4526449
IS91	+	4550495	4551727	4550840	4552072
IS91	+	4611085	4611981	4611429	4612325
IS1	-	4751112	4750425	4751456	4750781
IS110	-	4762919	4761939	4763263	4762283

IS3 ssgr IS51 (IS*1203*)	+	4871941	4873154	4872285	4873498
Tn3	+	4898795	4899355	4899139	4899699
IS3 ssgr IS51 (IS*1203*)	-	4945084	4944353	4945428	4944697
IS3 ssgr IS51 (IS*1203*)	+	5063999	5065212	5064343	5065556
IS256	-	5065409	5065215	5065753	5065559
IS630	-	5076748	5075903	5077092	5076247
IS1	-	5079228	5079082	5079572	5079426
IS4 ssgr IS50	-	5083152	5081884	5083496	5082228
IS66	-	5087964	5085478	5088308	5085822
IS66	+	5088048	5088838	5088392	5089182
IS200/IS605	-	5156369	5155770	5156713	5156114
IS200/IS605	+	5156392	5157276	5156736	5157620
IS3 ssgr IS51 (IS*1203*)	-	5158529	5157316	5158873	5157660
IS200/IS605	+	5158623	5158913	5158967	5159257
IS3 ssgr IS51 (IS*1203*)	+	5237462	5238771	-	-
IS91	+	5250312	5252018	5370412	5372088
ISNCY ssgr ISPlu15	+	5278376	5279332	5398445	5399401
IS21	-	5308582	5307845	5428651	5427914

Table S4: Summary of putative novel type IIG MTase encoding operon identified in the genomes of 7 complete *E. coli*

Strain	coordinates	Mtase	ATPase	Hypothetical1	Hypothetical2
95JB1	5233650..5243540	M.Eco95JB1IX	EC95JB1_03899	EC95JB1_03900	EC95JB1_03901
95NR1	5355040..5363617	M.Eco95NR1IX	EC95NR1_04072	EC95NR1_04073	EC95NR1_04074
11128	5255700..5264277	ECO111_5156	ECO111_5157	ECO111_5158	ECO111_5159
HS	340859..349415	EcHS_A0336	EcHS_A0337	EcHS_A0338	EcHS_A0339
i2	4888341..4897008	I02_4874b	I02_4875	I02_4876	I02_4877
i14	4888341..4897008	I14_4874b	I14_4875	I14_4876	I14_4877
EC958	4142001..4150509	EC958_4080	EC958_4081	EC958_4082	EC958_4083

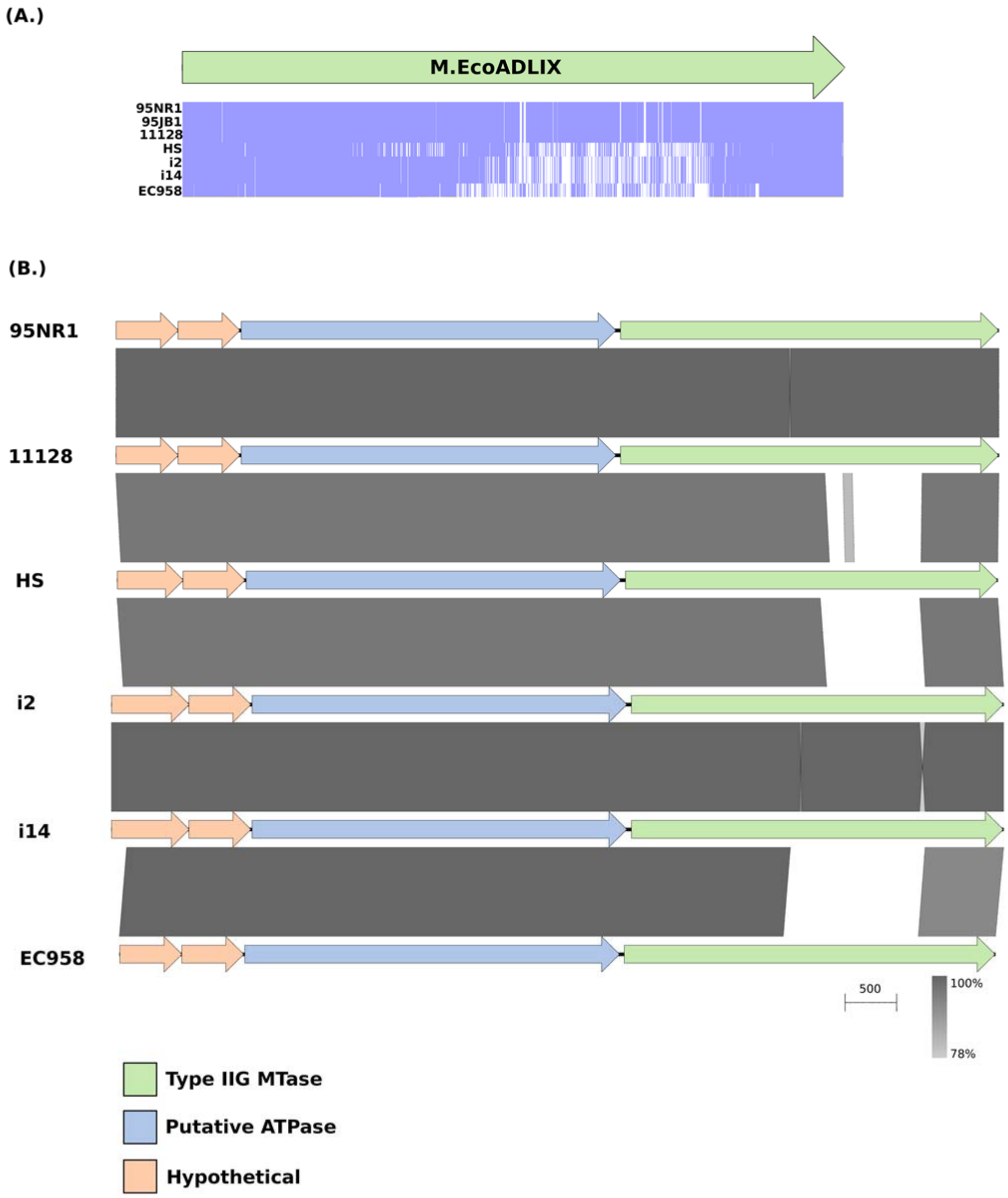

Figure S1: Comparison of the type IIG encoding operon from the genomes of 7 *E. coli.*
(A.) Amino acid comparison of type IIG MTase from 95NR1, 95JB1, 11128, HS, i2, i14 and EC958. Blue shading represents regions of homology present in two or more strains. Protein sequences were aligned using TCoffee[1] and visualised using Jalview[2]. (B.) Pairwise nucleotide comparison of the type IIG encoding operon in 95NR1, 1128, HS, i2, i14 and EC958. Genes are represent by arrows coloured as per the legend. Grey shading represents

regions of homology between strains from 78%-100% nucleotide sequence identity. The highly variable region present in the type IIG MTase of each strain is clearly visible.

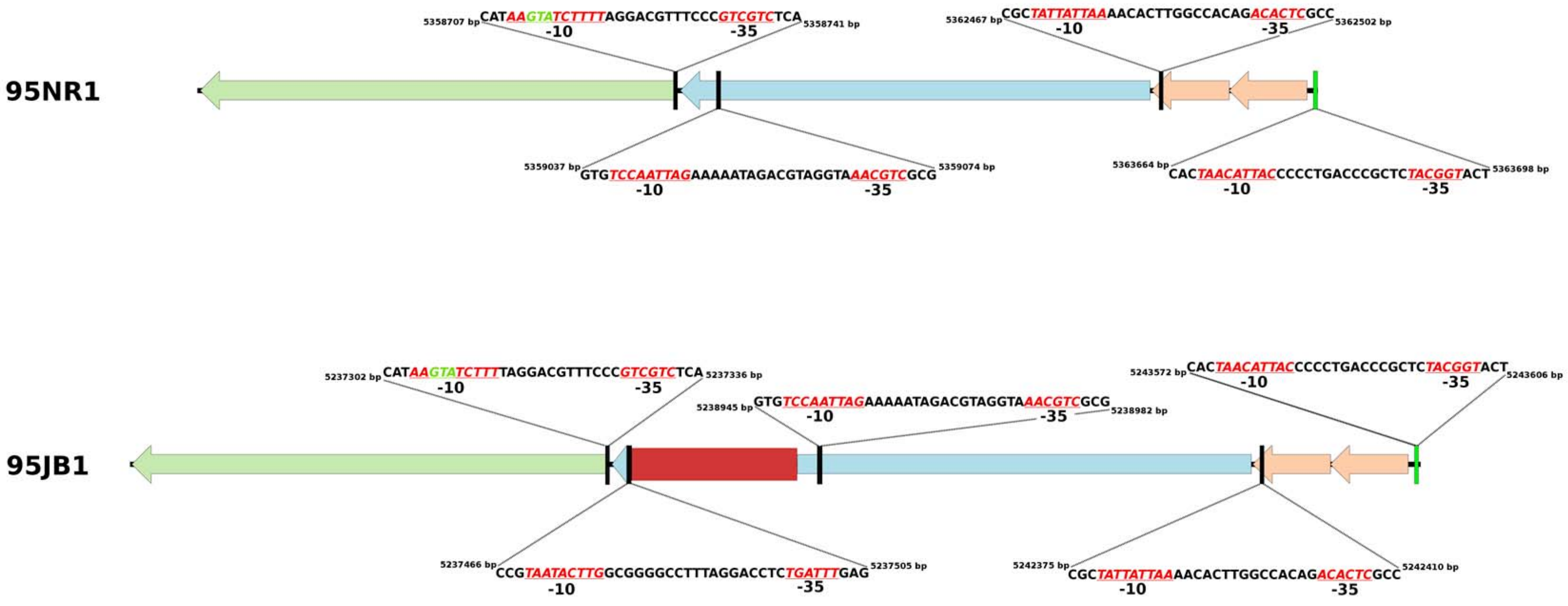

Figure S2: Schematic representation of the type IIG encoding operon of 95NR1 (top) and 95JB1 (bottom). Genes are represented by coloured arrows (green=MTase, blue=ATPase, orange=hypothetical) and the IS*1203*-like insertion into EC95JB1_03899 by the red rectangle. Predicted transcriptional start sites are represented by coloured bars (green=primary TSS, black=Alternate TSS). Popouts display the sequence of the TSS with the -10 and -35 boxes indicated in red.

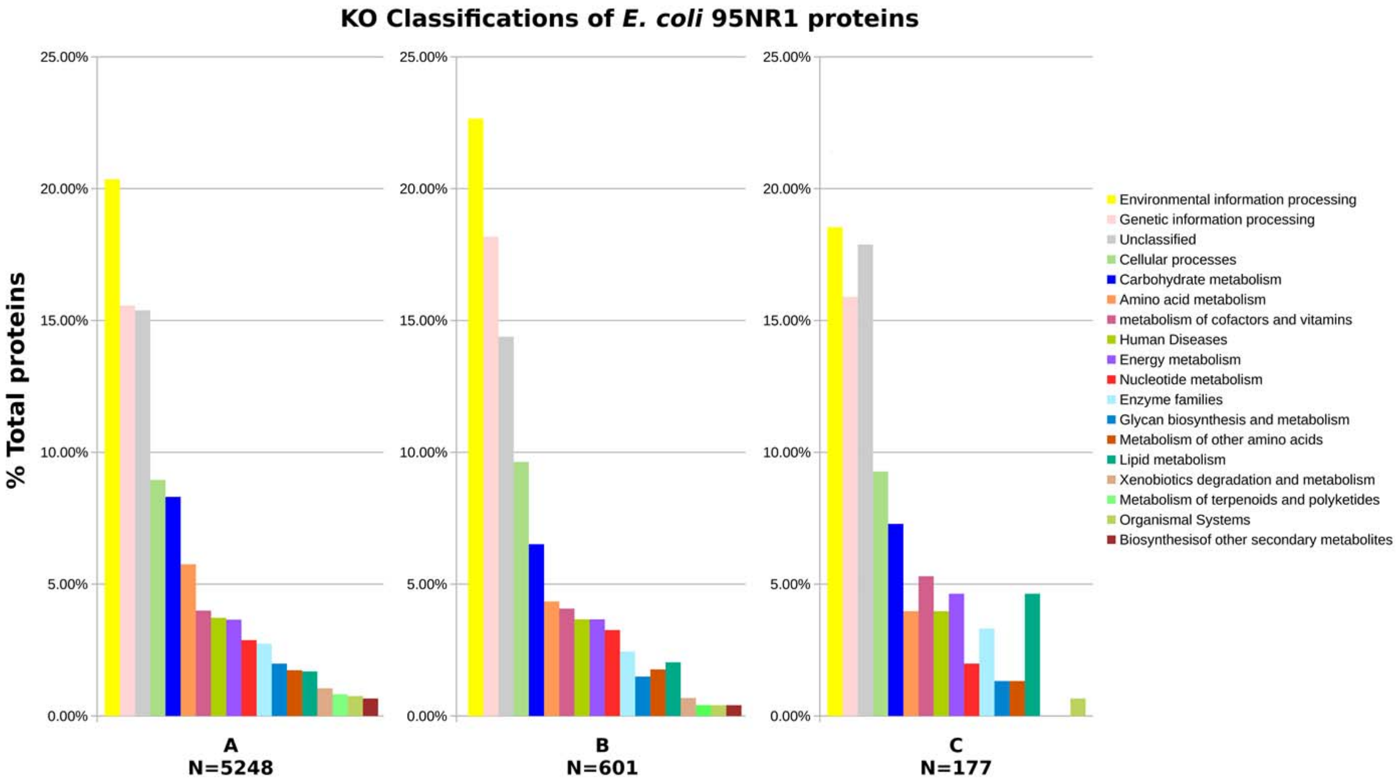

Figure S3: KEGG functional classification of 95NR1 proteins. The percentage of proteins belonging to a given KO category are listed for: A) all 5248 protein coding genes identified in 95NR1 for which COG categories could be assigned; B) all 601 protein coding genes identified as having a methylated CRARCAG motif within 300 bp of their respective start codons; C) all 177 protein coding genes identified as having a methylated CRARCAG motif within 300 bp of their respective start codons which lies in a predicted promoter region.

References for supplementary material

1 Magis, C. *et al.* T-Coffee: Tree-based consistency objective function for alignment evaluation. *Methods in molecular biology* **1079**, 117-129, doi:10.1007/978-1-62703-646-7_7 (2014).

2 Waterhouse, A. M., Procter, J. B., Martin, D. M., Clamp, M. & Barton, G. J. Jalview Version 2--a multiple sequence alignment editor and analysis workbench. *Bioinformatics* **25**, 1189-1191, doi:10.1093/bioinformatics/btp033 (2009).
